# Collaborative cytometric inter-laboratory ring test for probiotics quantification

**DOI:** 10.3389/fmicb.2023.1285075

**Published:** 2023-11-10

**Authors:** Peter Lüttge Jordal, Marcos González Diaz, Carlotta Morazzoni, Serena Allesina, Daniele Zogno, Daniela Cattivelli, Serena Galletti, Elena Guidesi, Jean-Pol Warzée, Marco Pane

**Affiliations:** ^1^SBT Instruments A/S, Herlev, Denmark; ^2^Probiotical Research, Novara, Italy; ^3^AAT – Advanced Analytical Technologies, Fiorenzuola d’Arda, Italy; ^4^European Scientific League for Probiotics, Brussels, Belgium

**Keywords:** probiotics, culture-independent, fluorescence flow cytometry, impedance flow cytometry, electrical impedance spectroscopy flow cytometry (EIS-FC), dormant, viable but non-culturable

## Abstract

**Introduction:**

Probiotics are live microorganisms that, when administered in adequate amounts, confer a health benefit on the host. From this definition, accurate enumeration of probiotic products is a necessity. Nonetheless, this definition does not specify the methods for assessing such viability. Colony forming units is the *de facto* gold standard for enumerating viable in probiotic products. The notion of microbial viability has been anchored in the concept of cultivability, which refers to a cell’s capacity to replicate and form colonies on agar media. However, there is a growing consensus that the term “viability” should not be exclusively tied to the ability to cultivate cells. For example, bacterial cells can exist in a Viable But Non-Culturable (VBNC) state, characterized by the maintenance of characteristics such as membrane integrity, enzymatic activity, pH gradients, and elevated levels of rRNA, despite losing the ability to form colonies.

**Methods:**

Herein we present the results of a collaborative inter-laboratory ring test for cytometric bacterial quantification. Specifically, membrane integrity fluorescence flow cytometry (FFC) method and the newer impedance flow cytometry (IFC) method have been used. Both methods interrogate single cells in solution for the presence of intact membranes. FFC exploits fluorochromes that reflect the presence or absence of an intact membrane. IFC probes membrane integrity in a label-free approach by detecting membrane-induced hindrances to the propagation of electricity.

**Results:**

A performance ring-test and comparison design on the FFC method showed that the method is robust against the exchange of equipment, procedures, materials, and operators. After initial method optimization with assessments of rehydration medium, wake-up duration, and phase shift gating on the individual strains, the IFC method showed good agreement with the FFC results. Specifically, we tested 6 distinct species of probiotic bacteria (3 *Lactobacillus* and 3 *Bifidobacterium* strains) finding good agreement between FFC and IFC results in terms of total and live cells.

**Discussion:**

Together, these results demonstrate that flow cytometry is a reliable, precise, and user-friendly culture-independent method for bacterial enumeration.

## Introduction

The concept of probiotics necessitates administering a specific quantity of viable bacteria to the consumer to yield health benefits (Hill, 2014). However, this quantity depends strongly on the type of analysis used. For over 125 years, the colony forming unit (CFU) has been the favored method for microbial enumeration (USP, 2021). In fact, CFUs are regarded as the probiotic industry’s analytical quantification gold standard (Weitzel et al., 2021).

Key advantages of the CFU method include its wide acceptance, technical simplicity, and ease of implementation. Yet, its limitations include low throughput, lengthy time-to-result (often exceeding 72 h of incubation), and low precision (Jackson et al., 2019). Using this method for certain bacterium types, like strict anaerobes, can also prove challenging. Furthermore, the CFU method has inherent blind spots. For instance, it requires bacterial proliferation for colony formation. Stressors known to potentially induce viable but non-culturable (VBNC) states in the production of probiotic bacteria are largely ignored in CFU analysis results (Emerson et al., 2017; Fiore et al., 2020; Foglia et al., 2020; Wendel, 2022). Another disadvantage is its inability to detect dead bacteria, making it inapplicable for postbiotic products constituted by inactivated bacteria.

Traditionally, viability has been gauged by cultivability, i.e., the ability to divide and form colonies, a principle originating from Robert Koch’s initial landmarks. However, recent suggestions advocate for a broader viability definition to include all metabolically active microbes or those with intact membranes (Breeuwer and abee, 2000). This extended definition permits the use of culture-independent techniques such as flow cytometry, and PCR- or FISH-based methods for viability assessment (Davis, 2014; Wendel, 2022).

Flow cytometry is emerging as a more extensive microbiological characterization method, capable of detecting and quantifying colony-forming, VBNC, and dead bacterial states (Chiron and Tompkins, 2017). It overcomes the CFU method’s limitations by offering real-time results and improved precision, detecting up to millions of objects with high sensitivity (Chiron and Tompkins, 2017; Fiore et al., 2020). As such, flow cytometry is gaining popularity as a speedy alternative for profiling microorganisms, including probiotics (Lahtinen et al., 2005; ISO, 2015; Jackson et al., 2019).

**Figure 1:**
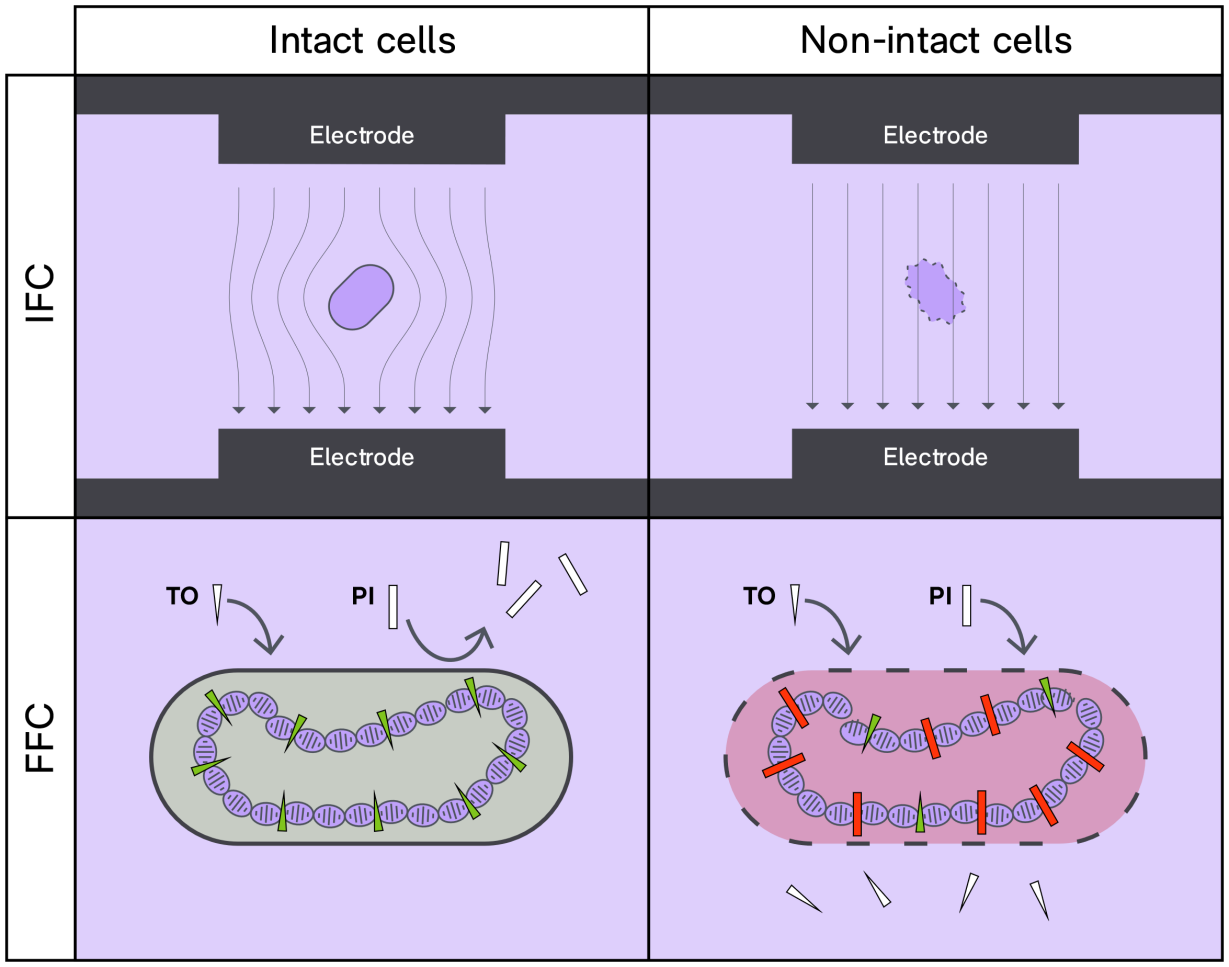
Interrogation of membrane intactness status by IFC and FFC techniques. With the IFC technique an intact lipid membrane will impose a pronounced hindrance to electricity that differs from that of non-intact bacteria. For FFC, membrane intactness is probed by differential penetrance to charged DNA-binding fluorophores. Thiazole orange (TO) can penetrate intact cell membranes and upon binding to DNA, TO will fluoresce green when excited at 488 nm. Contrarily, propidium iodide (PI) enters cells and bind to DNA if the membrane is compromised; PI will fluoresce red when excited at 488 nm.

Flow cytometry relies on the premise of studying individual cells within a heterogeneous population. In Fluorescence Flow Cytometry (FFC), membrane integrity stains are commonly used to discern the live/dead status of bacteria (Figure 1). Certain dyes, such as propidium iodide (PI), can enter bacteria with compromised membranes, while other membrane-diffusible stains, like SYTO-9, SYTO-24, and thiazole orange, can permeate bacteria independent of membrane status. These fluorophores change their emission properties when bound to bacterial DNA. The concentration of active (or viable) bacteria is calculated by deducting the dead proportion from the total cells (ISO, 2015; Wilkinson, 2018).

While FFC holds clear advantages over CFUs, it also has limitations, such as complex staining procedures, the use of potentially carcinogenic substances, high initial investment costs, and the need for skilled operators (Davey, 2011; Zand et al., 2021). Furthermore, there is a concern that stains, solvents, and penetration enhancers could affect the bacteria’s membranes (Chitemerere and Mukanganyama, 2014; Nescerecka et al., 2016; Deng et al., 2020).

Recently, Impedance Flow Cytometry (IFC) or Electrical Impedance Spectroscopy (EIS-FC) has been introduced as a label-free technique for enumerating and characterizing viable bacteria (Clausen et al., 2018; Bertelsen et al., 2020; Modena and Hierlemann, 2021). IFC, an adaptation of the Coulter counter principle, is a multiparametric method to analyze cells in suspension (Coulter, 1953; Modena and Hierlemann, 2021). Briefly, IFC uses a narrow microfluidic channel and electrode sets in contact with the liquid. The passage of an object through the electric field results in a slight impedance change, which is used to obtain information on the object’s size, membrane integrity, and intracellular content (Figure 1). By analyzing the change in impedance, one can determine whether the bacterium’s membrane is intact or compromised (Sun and Morgan, 2010; Clausen et al., 2018; Bertelsen et al., 2020; Bertelsen, 2021).

In this study, we aimed to use a ring-test design to establish robust FFC-based active and total bacterial concentrations for six probiotic bacteria and subsequently compare these results with the label-free IFC technique. To our knowledge, the present study represents the first peer-reviewed comparison of FFC and IFC for bacteria enumeration.

## Materials and methods

### Species information

Probiotic bacteria used for the present study were provided by Probiotical and are referenced with their internal identifiers. *Lactobacilllus* species were *Lactiplantibacillus plantarum* (ID 091), *Lactocaseibacillus rhamnosus* (ID 1697), *Lacticaseibacillus casei* (ID 1872), *Bifidobacterium breve* (ID 1747), *Bifidobacterium longum* (ID 1152), and *Bifidobacterium animalis* subsp. *lactis* (ID 1518). In addition, *Lactocaseibacillus rhamnosus* PB01 (DSM 14870) was provided from Deerland probiotics. This strain was not part of the ring test experiments but was included in this publication because it showed the most profound changes in phase shift and amplitude distributions during the wake-up experiments.

### Cultivation

To optimize live/dead classification of the given strains, cultures were maintained in an incubator (37°C, 200 RPM) throughout the lag-, exponential-, stationary-, and death phase. Inoculation was done by transferring 1 μL of 1:10 (g/g) stomacher homogenate to a ready-made MRS broth vial (Bio-Rad laboratories inc, cat. no. ##3554488). Tubes were placed horizontally for efficient agitation.

Plate counts (see Supplementary Material) were performed in accordance with existing ISO methods: ISO 29981 IDF 220 for *Bifidobacteria* spp. and 27,205 IDF 149 – ISO 7889 IDF 117 for *Lactobacillus* spp. Briefly, an amount of 4.0–5.0 g of sample was serially diluted in peptone saline water solution. The appropriate dilutions were plated by inclusion technique on TOS-propionate agar medium or De Man, Rogosa and Sharpe agar according to the genera and relative ISO method. Plates were then incubated in anaerobic jar at 37°C and colonies counted after 72 h of incubation.

### Sample preparation

All freeze-dried samples were stored at −20°C in sealed aluminum sachets until the time of analysis. Samples were reconstituted at 1:10 (g/g) in either buffered peptone water or PBS (for the FFC technique) or MRS broth (for the IFC technique) and subjected to stomacher homogenization (Seward stomacher model 400, 260 RPM, 4 min). For the IFC technique, the sample was re-homogenized after 30 min (stomacher, 260 RPM, 1 min).

### Fluorescence flow cytometry

FFC was performed in a ring test design at three different companies: Probiotical QC laboratory conducted experiments with 2 different operators in 2 different QC laboratories using a FACS Calibur instrument (Becton Dickinson). Probiotical R&D laboratory conducted experiments with 2 different operators in 2 different R&D laboratories using a Cytoflex instrument (Beckman Coulter). AAT-Advanced Analytical Technologies conducted experiments in the same laboratory with two different operators using an Attune NxT instrument (Thermo Fisher). The FACS Calibur uses analogue technology with hydrodynamic focusing and reference beads as internal standard to calculate absolute concentrations of bacteria. The Beckman Cytoflex is a digital instrument with hydrodynamic focusing and absolute concentrations based on volumetric counting instead of internal standards. Finally, the Thermo Fisher Attune NxT instrument uses acoustic focusing and reference beads as internal standard to calculate absolute concentrations of bacteria.

The BD Cell Viability Kit with liquid counting beads (BD Biosciences, Cat. no. 349483) was used. Cell staining was performed according to ISO 19344: IDF 232 (2015). Briefly, 100 μL of a diluted suspension containing approximately 10^5^–10^6^ cells/mL in buffered peptone water was added to 835 μL of PBS. Then 10 μL of PI (prior diluted in water at 0.2 mmol/L) and 5 μL of TO (42 μmol/L) were added to the dilution and the sample was vortexed. Stained sample was incubated for 15 min at 37°C in the dark. For the flow cytometers without volumetric counting, the counting beads suspension was gently vortexed for 30 s and then 50 μL was added to the cell suspension for a final volume of 1 mL. For Cytoflex instruments no counting beads were added because the concentration is based on the defined sample volume taken from the needle. In this case the volume of PBS for the final dilution was 885 μL instead of 835 μL.

For the Attune NxT instrument, cell staining was performed according to the ISO 19344:2015 IDF 232:2015, protocol B. Briefly, cells were diluted in decimal serial dilutions in PBS to obtain about 10^5^ cells/mL: 100 μL of this final dilution was added to 880 μL of PBS. Then 10 μL of PI (prior diluted in H_2_O at 0.2 mmol/L) and 10 μL of Syto24 (prior diluted in H_2_O at 0.1 mmoL/L) were added to the dilution and the sample was vortexed. The stained sample was incubated for 15 min at 37°C in the dark. Before analysis, the counting beads were used as internal control (ThermoFisher Scientific, Cat. No. C36950) was gently vortexed for 30 s and then 50 μL was added to the cell suspension for a final volume of 1,050 μL.

#### FACSCalibur acquisition settings

The FACScan FACSCalibur cytometer (BD FACSCalibur Software; Becton Dickinson, San Jose, CA) was equipped with 488 nm argon laser excitation and CellQuest software. An SSC-H (Side Scatter) threshold was used for microbial cells. Cells were gated using forward versus side scatter (FSC-H vs. SSC-H). Thiazole Orange (TO) fluoresces primarily in the FL1 channel and Propidium iodide (PI) fluoresces primarily in the FL3 channel. The best discrimination of live and dead populations was on an FL1 versus FL3 plot. To exclude any false positive and negative results, reference control gating was generated on a fresh culture of *L. rhamnosus* GG; the fresh culture was representative of live population while the same culture after isopropanol treatment was used as reference for dead cell population. Live sample was stained only with TO while dead sample was stained with PI.

#### Attune NxT acquisition settings

The Attune NxT Acoustic Focusing Cytometer (Thermo Fisher) was equipped with 488 nm laser excitation. An SSC-H (Side Scatter) and FSC-H (Forward Scatter) thresholds were used for microbial cells. Cells were gated using forward versus side scatter (FSC-H vs. SSC-H). Syto 24 fluoresces primarily in the BL-1 channel and Propidium iodide (PI) fluoresces primarily in the BL-3 channel. The best discrimination of live and dead populations was on an BL-1 versus BL-3 plot.

#### Cytoflex acquisition settings

The CytoFLEX cytometer (Beckman Coulter srl) was equipped with 488 nm laser excitation and CytExpert software. An SSC-H (Side Scatter) and FSC-H (Forward Scatter) thresholds were used for microbial cells. Cells were gated using forward versus side scatter (FSC-H vs. SSC-H). The best discrimination of live and dead populations was on an FL1 versus FL3 plot.

### Impedance flow cytometry

IFC was done in a single laboratory (SBT Instruments) by a single operator using a BactoBox^®^ HW version 7.4, SW version 2023.04. To be within the linear measurement range, each 1:10 (g/g) stomacher homogenate was diluted by two consecutive DF 201 dilutions in BactoBox diluent (50 μL sample added to 10,000 μL 1:9 PBS). Samples were analyzed immediately after preparation of each dilution series. Replicates were based on fresh dilution series of the stomacher homogenates. At least three replicates with fresh dilution series were prepared for each species.

### Microscopy

An LS620 fluorescence microscope (Etaluma) equipped with an Olympus 60× long-working distance objective was used to obtain information on the presence of single-cell suspensions as well as live/dead information based on membrane integrity. Samples were prepared by depositing 2 μL sample on an objective glass and subsequently pressing the droplet flat with a cover slip and the posterior end of a plastic Pasteur pipette. Membrane intactness was evaluated using a combination of phase contrast (total objects), SYBR-green I (for total bacterial, Thermo Fisher cat. No. #S7563) and thiazole red, TO-PRO-3 (for impaired membrane, Biotium cat. No. #40087). Bacteria were stained in the dark in 1× PBS using 1:10,000 dilution of the stock concentration of SYBR-green I and 10 μM TO-PRO-3 for 15 min. Live/dead fluorescence thresholds were adjusted by analyzing 100% live samples and samples killed with 70% denatured alcohol for 15 min.

### Statistical analysis

The “Data Analysis” plug-in for excel was used to assess the statistical significance of variance of the mean with the single-factor and Nested ANOVA tool using an alpha value of 0.05. Probability values (*p*-values) below 0.05 were considered statistically significant. Scatter charts were prepared in Graphpad prism.

## Results

With membrane integrity as a proxy for bacterial viability we set out to investigate two flow cytometry platforms for enumeration and live/dead characterization of freeze-dried probiotic bacteria. Membrane-integrity FFC is already an established technique for bacteria and the method parameters are well-defined for routine in-house quality as outlined in ISO 19344. Contrarily, for the IFC technique, the present study on freeze-dried probiotics is the first of its type and therefore method optimization was needed prior to determining actual bacterial concentrations and live/dead ratios. Four major learnings were realized for the IFC technique:

- Concentrations exceeding 500,000 total particles/mL are required to obtain reliable live/dead ratios.
- Some probiotic bacteria do not adhere to the default IFC rulesets and require custom gating for accurate live/dead classification.
- Nutrient-rich media such as MRS broth are required as rehydration medium before conducting measurements.
- A wake-up period of 30–60 min is necessary to rehydrate and obtain normal impedance fingerprints before IFC measurements can be made.

### IFC analyses should be performed at concentrations exceeding 500,000 total particles/mL

It is generally recommended to conduct analyses at a particle concentration ranging between 500,000 to 5,000,000 total particles per mL. This recommendation is based on two fundamental reasons.

Firstly, precision in particle detection statistics tends to improve with the detection of a larger number of events, provided that the detector element does not become oversaturated. This means that gathering as many data points as possible helps to ensure that the analysis is accurate and reliable.

Secondly, the operation of the IFC instrument involves a peristaltic pump, whose head rollers can occasionally release microplastic particles from the peristaltic tubing. At low bacterial concentrations, longer measurement times, such as three minutes, are typically employed. Because the sample is constantly recirculated during this process, the microplastic particles can be detected as non-conductive objects, artificially inflating the concentration of non-bacterial objects. These microplastic objects are typically observed at approximately 1 radian, a range outside that of the dead bacterial phase shift. However, they are still included in total concentrations, which can potentially lead to an underestimation of the live/dead ratio. To avoid this, maintaining a high bacterial concentration is necessary to reduce the proportion of these non-conductive, non-bacterial objects in the sample.

### Custom gating is needed for some probiotic bacteria

The IFC method typically employs a default ruleset for live/dead classification, specifically defining the intact cell region with a lower and upper value for the 7 MHz phase shift angle. Our initial analysis using these default parameters yielded an excellent agreement between FFC and IFC for two strains, *L. plantarum* and *B. lactis*, as will be discussed later. However, for the remaining strains, the Intact Cell Concentration (ICC) determined by IFC were frequently about 50% lower than the Active Fluorescent Units (AFU) determined by the FFC method.

**Figure 2:**
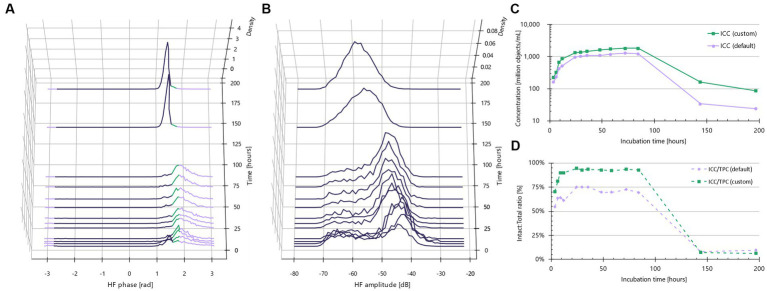
Custom gating is needed for *Lacticaseibacillus casei*. **(A)** Normalized phase shift distributions plotted as a function of incubation time. Lavender color indicates default outside phase shift limits from −2.72 to +1.83 rad. Green color indicates lowering of default limits to +1.60 rad and violet color indicates other particles than intact cells, e.g., dead cells. **(B)** Normalized amplitude distributions plotted as a function of incubation time. **(C)** Growth curve for default intact cells (lavender) and custom-gated intact cells (green). **(D)** Ratio between intact and total cells as a function of incubation time.

Recognizing that the default IFC gating thresholds were suboptimal for these four strains, we sought to customize the phase shift thresholds. To achieve this, we created sample sets composed almost entirely of live bacteria and other sample sets consisting of almost entirely dead bacteria, as illustrated with *L. casei* (Figure 2).

Both live and dead sample sets were derived from a straightforward batch inoculation growth curve experiment conducted in MRS broth. Spectra representing 100% live bacteria were obtained by analyzing the bacteria during the mid- to late-exponential growth stage. For *L. casei*, these “100% live” timepoints occurred between 3.6–9.0 h (Figure 2A). At these points, the bacteria had yet to enter the death stage, making the initial presence of dead cells negligible. Fluorescence microscopy with SYBR Green I (total stain) and TO-PRO-3 (impaired membrane) confirmed the presence of 100% intact bacteria at these timepoints (results not shown).

Similarly, impedance spectra of 100% dead cells were obtained by analyzing the cultures when the bacteria had fully entered the death stage, for *L. casei* this corresponded to 6 and 8 days of incubation, i.e., 144 and 191 h, respectively (Figure 2A). TO-PRO-3 staining clearly indicated that these cultures consisted almost entirely of dead bacteria.

Upon examining the phase shift distributions for the 100% live incubation times, we found a significant proportion of the objects consistently in the non-intact range from 1.60 rad to 1.83 rad. This resulted in an intact:total plateau at 75% (Figure 2D, violet curve). The intersection between the 100% live and 100% dead cultures was found at 1.6 rad. By reducing the upper phase shift threshold to 1.6 rad, the intact:total ratio now approached 100% for the high viability incubation times, and remained close to 0% when the culture had entered the death stage (Figure 2D, green curve).

Similar growth curve experiments were conducted to optimize gating for *L. rhamnosus*, *B. breve*, and *B. longum*. As will be demonstrated later, these refinements significantly improved the correlation between Active Fluorescent Units (AFU) and Intact Cell Concentrations (ICC), moving from a roughly 50% correlation to a near 1:1 agreement.

### MRS medium is needed for proper rehydration of *Lactobacillus* species

**Figure 3:**
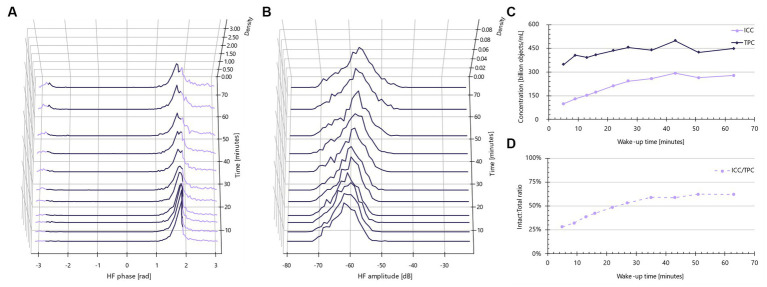
Impedance signatures for *Lacticaseibacillus rhamnosus* PB01 change during wake-up in MRS medium. **(A)** Normalized phase shift distributions plotted as a function of wake-up time. Lavender color indicates default intact cell limits. Violet color indicates other particles than intact cells, e.g., dead cells. **(B)** Normalized amplitude distributions plotted as a function of incubation time. **(C)** Intact cell and total particle concentration plotted as a function of wake-up time. **(D)** Ratio between intact and total cells as a function of incubation time.

The ISO FFC method for probiotic bacteria (ISO, 2015) advises the use of phosphate-buffered saline (PBS) as a diluent in the membrane-integrity assay protocol (assay B). In contrast, for the IFC method, it quickly became apparent that MRS broth was necessary, particularly for *Lactobacillus* strains (Figure 3). Upon reconstitution in MRS, over time, a portion of the bacteria demonstrate a noticeable shift to the right, indicating a trend towards more electrically conductive properties. In other words, the primary non-conductive population at approximately 1.8 rad after 5 min of ‘wake-up’ time gradually decreases, while the proportion of objects in the lavender region expands (Figure 3A). Concurrently, the high frequency (HF) amplitude rises, suggesting an increase in object size and/or enhanced electrically conductive properties (Figure 3B). This change leads to an increase in intact cell concentrations over time (Figure 3C), likely attributable to the rehydration of freeze-dried, anhydrous cells. Comparatively, the total particle concentration remains relatively stable over time, suggesting no cellular division. Viewed as a proxy for the live/dead ratio, the ‘wake-up’ period appears to stabilize after approximately 35 min.

**Figure 4:**
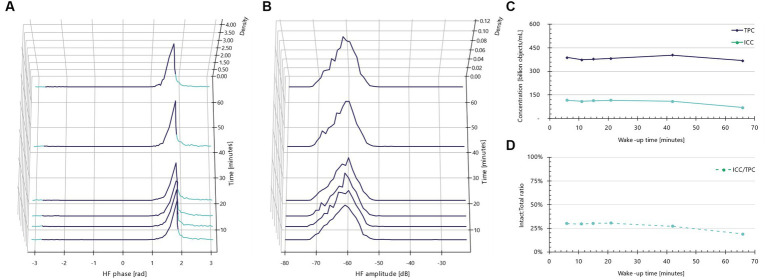
Impedance signatures for *Lacticaseibacillus rhamnosus* PB01 do not change in BPW. **(A)** Normalized phase shift distributions plotted as a function of wake-up time. Teal color indicates default intact cell limits. Violet color indicates other particles than intact cells, e.g., dead cells. **(B)** Normalized amplitude distributions plotted as a function of incubation time. **(C)** Intact cell and total particle concentration plotted as a function of wake-up time. **(D)** Ratio between intact and total cells as a function of incubation time.

When the same rehydration experiment is conducted with buffered peptone water (BPW), the phase shift populations and amplitudes (Figure 4) do not display the same morphological and/or electrical changes observed with rehydration in MRS broth. Instead, the phase shift distributions remain unchanged over time, barring a broadening of the left shoulder at the 66 min mark (Figure 4A). Furthermore, more low-amplitude objects appear at later time points (Figure 4B). Cumulatively, these observations indicate a time-dependent increase in presumable dead cells with low conductivity and smaller amplitudes. The decline in intact cell concentrations over time is evident (Figure 4C), particularly after about 40 min of incubation. This trend is further supported by the decreasing intact-to-total ratio (Figure 4D); rehydration in BPW results in a drop from approximately 25 to 20%, compared to an increase from roughly 25 to 60% when rehydrated in MRS broth. Therefore, MRS not only prevents lysis of freeze-dried *Lactobacillus* species but also allows the cells to kickstart their metabolism.

In summary, for reliable live/dead assessments using the IFC method, it is crucial to use MRS broth as a rehydration medium and to allow for a ‘wake-up’ time of 30–60 min. The suitable rehydration duration varies depending on the specific species. As the total particle concentration remains stable throughout this period, there’s no risk of overestimation due to cell division. An advantage of the IFC method is that the results from ‘wake-up’ experiments are instantly available. This allows for the real-time monitoring of morphological and membrane-related changes.

### FFC and IFC results are in good agreement

Following the initial optimization of the IFC method, the results were compared with the findings from the FFC technique. It’s crucial to note that while the IFC analyses were conducted in a single laboratory, the FFC analyses utilized a ring test design, with tests performed at three distinct companies each using a different type of cytometer: Becton Dickinson’s FACS Calibur (using beads as an internal standard), Beckman Coulter’s Cytoflex (which performs volumetric absolute counts), and Thermo Fisher’s Attune (which uses an acoustic focusing method). Consequently, greater variability is observed in the FFC with an uncertainty of 0.24 and 0.20 for Active Fluorescent Units (AFU) and Total Fluorescent Units (TFU) respectively, making it more meaningful to compare the averages of the two types of membrane-integrity flow cytometry.

Moreover, it was possible to evaluate the ring-test performances across three different labs using substantially different FFC equipment which differentiate according to the references and sampling technologies (volumetric absolute counts vs. beads used as reference and hydrodynamic focusing versus acoustic focusing methods) with an overall reproducibility (S_R_) of 0.12 and 0.10 for AFU and TFU respectively, that are lower than those defined in the ISO 19344:2015 (S_R_ reported as 0.16 and 0.134 for AFU and TFU respectively). The *Z*-scores calculated for the different laboratories that performed the FFC analysis were all lower than 2.

**Figure 5:**
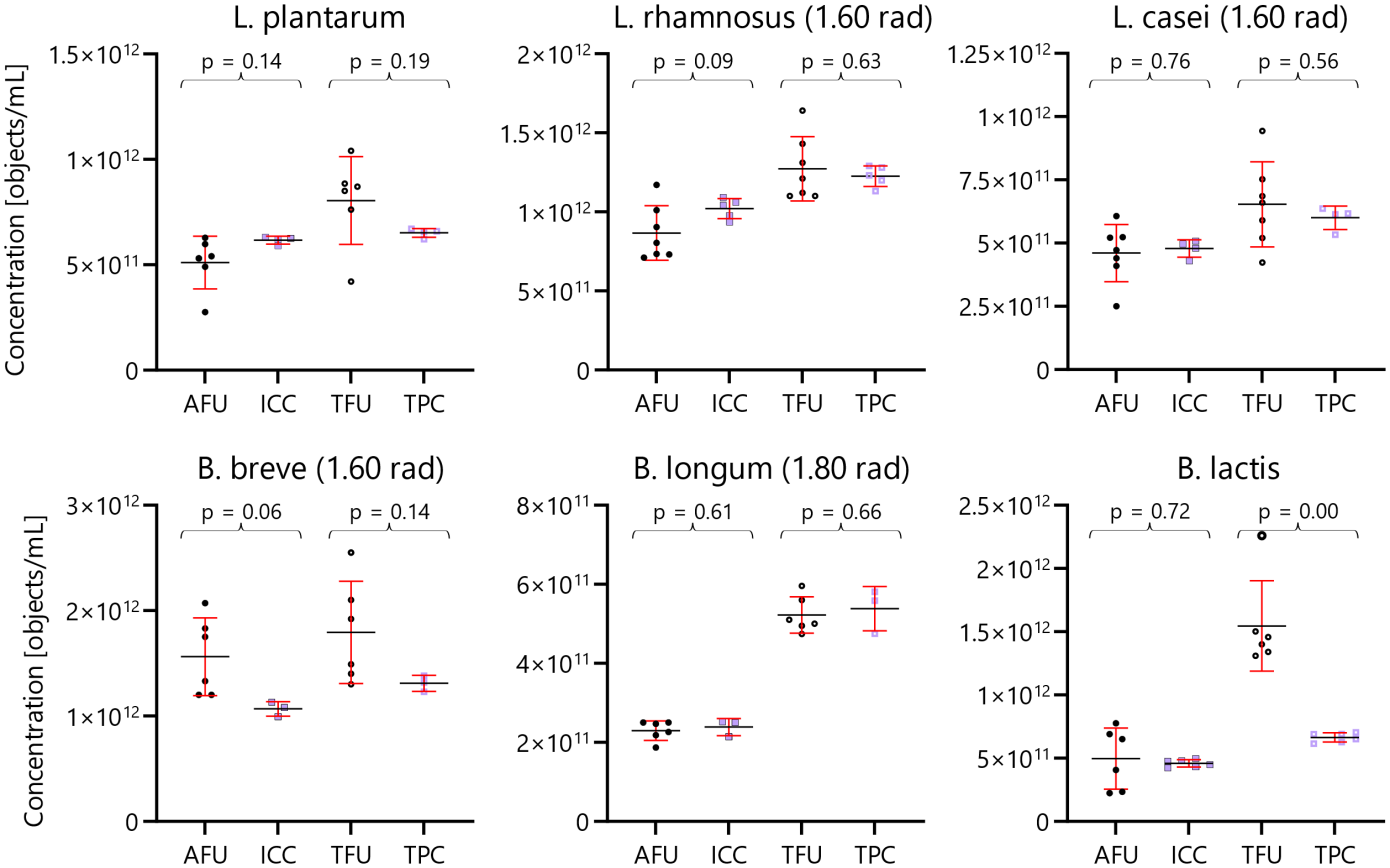
Head-to-head comparison of FFC and IFC techniques. Scatter charts for three *Lactobacillus* and three *Bifidobacterium* species. FFC measurements are shown with black circles, while IFC measurements are shown with lavender boxes. Active fluorescent units (AFU) and intact cell concentration (ICC) are depicted with filled data points, while total fluorescent units (TFU) and total particle concentrations (TFU) are shown with hollow data points. Average concentrations are depicted with black, horizontal bars while standard deviation is depicted with red error bars. Results of ANOVA tests for each AFU:ICC and TFU:TPC comparison is shown with brackets and *p*-values above each set of data points. Note that the FFC results were performed with a ring test design, while IFC analyses were done in a single lab.

Broadly, the results in this article from FFC and IFC techniques align well (Figure 5). This consistency is seen when comparing the active fluorescent units (AFU) from the FFC technique with the intact cell concentration (ICC) from the IFC technique with an estimated overall uncertainty of 0.12 – all tested species did not show statistically significant difference. Similarly, a good agreement is also evident when comparing total fluorescent units (TFU) and total particle concentration (TPC) derived from the FFC and IFC methods with an estimated overall uncertainty of 0.23 – five out of six tested species did not show statistically significant difference.

The average concentrations of *L. casei* and *B. longum* show exceptional agreement, being virtually identical. The comparison does not reveal statistically significant differences (*p* < 0.05) except for one instance, involving *B. lactis* where the TFU is approx. Twice the result of TPC. For this species, the amplitude was low (centered at −65 dB). Typically, dead cells shrink in size and potentially these exceedingly small objects could be hidden by the background noise. Another potential explanation for the discrepancy between TFU and TPC for *B. lactis* might be that the FFC gating includes objects other than dead cells.

In summary, the six species of probiotic bacteria analyzed yielded highly comparable bacterial concentrations for live and dead cells when comparing the FFC and IFC methods. Concentrations from the plate count technique are available in the Supplementary Material. Plate counts differ in the detection principle as it relies on cultivability opposed to membrane integrity probed by FFC and IFC. Nonetheless, agreement between AFU, ICC and CFU is within 50% for all species except for *Bifidobacterium animalis* subsp. *lactis*.

## Discussion

In this study, our goal was to evaluate the concordance between two flow cytometry methods, FFC and IFC. Both techniques assess bacterial viability by examining the integrity of the lipid membrane, serving as a key indicator of the live/dead status of bacteria.

### The need for custom IFC classification parameters

In this study, four out of the six examined probiotic bacterial strains required tailored classification parameters for precise live/dead determination. The standard IFC classification ruleset is predicated on the analysis of five actively growing cultures, with diverse Gram statuses and morphologies, including *E. coli* ATCC 8739 (rod-shaped, Gram-negative), *A. baumannii* ATCC 12457 (coccobacillus, Gram-negative), *S. epidermidis* ATCC 12228 (coccoid, Gram-positive), *K. aerogenes* ATCC 16048 (rod-shaped, Gram-negative), and *L. innocua* ATCC 33090 (rod-shaped, Gram-positive) (SBT Instruments, 2023). When cultured under ideal growth conditions, all these species are approximately 0.5 μm wide.

Conversely, the four probiotic bacteria requiring customized classification parameters appeared thinner when inspected using phase-contrast microscopy. This observation implies a larger membrane surface area relative to their cytoplasmic volume. Considering the hydrophobic, non-charged properties of the membrane’s interior, which hinders the propagation of current at 7 MHz, coupled with the cytoplasm’s ion-rich nature that excellently propagates electricity, it can be concluded that thin bacteria will typically propagate current less effectively than the default classification IFC ruleset accounts for.

As a result, all four bacteria needing custom gating required a decrease in the upper phase shift threshold to account for their less electrically conductive properties. Importantly, the optimization of gating is a one-time engineering effort per strain, which means routine post-analysis gating is not necessary. In general, we recommend investigating the need for custom gating by performing IFC measurements in conjunction to a simple shake flask experiment. Subsequently gating parameters can be established by comparing the phase shift distribution for the late-stage exponential culture (approx. 100% live) with an extensively aged or alternatively boiled culture (approx. 100% dead).

### Wake-up in growth medium

Significant shifts in the phase and amplitude distributions, particularly for *Lactobacillus* species, were observed when the lyophilized powders were rehydrated using a rich MRS broth. To the best of our knowledge, this study is the first to monitor this reactivation phenomenon in real time using the IFC technique. This phenomenon was not seen when buffered peptone water was used. A review of scientific literature reveals that this methodology aligns with the USP’s recommendation for the cultivation-based enumeration of probiotic bacteria. As per the USP, the sample should be dissolved in MRS broth, homogenized using a blender or stomacher, pre-incubated at room temperature, and re-homogenized before analysis (USP, 2019).

Furthermore, the ISO 19344 protocol for DiOC2 favors the use of a rich rehydration medium like MRS or M17 broth (in the case of *S. thermophilus*) to activate the cells. It also suggests a 30 min pre-incubation or “wake-up” period at 30°C for mesophilic bacteria and 37°C for thermophilic strains (ISO, 2015).

During the 0–60 min wake-up phase, no significant cell division was observed according to the total particle concentration. Increases in amplitude and shifts towards more conductive objects could be attributed to changes in object size and alterations in membrane or cytoplasm constitution. Microscopic evaluation of the *L. rhamnosus* PB01 strain during wake-up suggested an increase in object size over time (results not shown), thus the most plausible explanation for the changes in impedance properties is that the dehydrated bacteria are swelling in the nutrient-rich medium. Most wake-up intact:total curves stabilized within 30–40 min, making it feasible to complete the procedure within the 45 min permitted by the ISO 6887-1 method between sample rehydration and plating.

Curiously, the same wake-up effect was not observed for the *Bifidobacterium* species in MRS. Bifidobacteria are less oxygen-tolerant than *Lactobacillus* species (Charteris et al., 1997). The rehydration procedures were carried out in an ambient atmosphere where oxygen was present, which, we hypothesize, could have inhibited the activation of the bifidobacterial species. This theory aligns with the observation that *Lactobacillus* species demonstrated a short lag phase of a few hours, while the bifidobacterial lag phase extended to about 24 h. Currently, studies are underway to compare the wake-up effects of Bifidobacteria in rich MRS medium under both aerobic and anaerobic conditions.

### Comparative overview of FFC and IFC from a helicopter perspective

Flow cytometry significantly improves repeatability over traditional plate count methods, offering the additional benefit of distinguishing between live and dead bacteria and analyzing heterogeneous bacterial samples (Foglia et al., 2020; Michelutti et al., 2020; Tracey et al., 2023). The robustness of the FFC technique was reaffirmed in our ring test design, demonstrating its reliability against variations in operators and analytical instruments.

Both FFC and Impedance Flow Cytometry (IFC) deliver similar outcomes in assessing membrane integrity. However, differences are evident in the methods’ versatility and the requisite skill-level for instrument operation. FFC is versatile yet complex, with the ability to select from a wide range of stains and excitation parameters. This versatility is advantageous when exploring various aspects of viability, as exemplified in the ISO 19344 standard, which describes three proxies for viability (ISO, 2015). Moreover, FFC allows for fluorescence-assisted cell sorting and species-level discrimination using techniques like Fluorescent *in-situ* Hybridization (FISH) or strain-level discrimination with antibody-probes (Chiron and Tompkins, 2017). However, FFC’s precision and versatility come with higher costs and the need for extensive operator training, making it a common choice for end-point analysis in centralized quality control and research laboratories (Modena and Hierlemann, 2021).

In contrast, IFC is cost-effective, easy to implement, and practically operator-independent. Its compact size (30 × 30 × 20 cm) allows it to fit in a Laminar Air Flow (LAF) cabinet or an anaerobic chamber. IFC’s robustness is evident in its label-free technique, which only requires dilution of the primary sample. This simplicity contributes to its tight standard deviations with low coefficients of variations (CVs) ranging from 2 to 10%. Additionally, its quick sample preparation enables real-time measurements, as demonstrated with the wake-up data for *L. rhamnosus* PB01. However, IFC’s potential limitations include limited capacity to discriminate bacterial species in multispecies samples. Consequently, IFC is best suited for total counts or monoculture applications, where the primary requirements are to ascertain the concentration of viable bacteria and to determine the sample quality based on the live/dead ratio.

## Conclusion

Six single probiotic strains (3 lattobacilli and 3 bifidocateria) were evaluated using both Fluorescent Flow Cytometry (FFC) and Impedance Flow Cytometry (IFC) techniques, using membrane integrity as a proxy of bacterial viability. The ring-test design for the FFC technique included three different labs with substantially distinct FFC equipment differing with respect (i) volumetric absolute concentrations versus beads as a reference and (ii) with or without acoustic focusing. All laboratories have *Z*-scores less than 2 with data within the uncertainty defined by ISO 19344.

Once custom gating parameters were established for the IFC technique, a good agreement was observed between the two methods: 6 out of 6 strains did not show statistically significant difference when comparing FFC active fluorescence units to IFC intact cell concentrations. When comparing the FFC total fluorescent units with IFC total particle concentrations 5 out of 6 strains did not show statistically significant difference. In addition, this research represents the pioneering effort in utilizing impedance flow cytometry to observe the initial stages of rehydration kinetics. In line with the established procedures for membrane-potential-sensitive dyes such as DiOC_2_, a preliminary activation period of approximately 30 min in a nutrient-rich medium is essential to activate bacterial metabolism. Once this phase is completed, the proportions of live-to-dead cells can be accurately determined thereby improving assessment of the heterogeneity of bacterial populations in the sample. These results are extremely promising and further analysis on a bigger panel of different bacterial species is required to confirm these findings.

Probiotics quality is a key credibility factor for health care professionals and for consumers. Recently probiotics products have diversified to novel products containing, e.g., strictly anaerobic bacteria and inactivated bacteria (postbiotics). The emergence of these challenges poses significant analytical obstacles for the conventional gold standard plate counts used in probiotic product testing. Therefore, instrumentation and procedures need to be improved to assure reliable characterization and quantification of bacteria. Beside metagenomics and qPCR, flow cytometry proposes analysis opportunities of real and deeper efficiency as alternative or complementary to conventional microbiology (Warzée et al., 2021). This innovative and collaborative inter-laboratory approach for bacterial quantification is truly unique and plays a pivotal role in driving advancements in instrumentation within this field. Finally, we do reiterate that careful evaluation was done defining the measurand (Weitzel et al., 2021) which in our setting was an industrial derived sample of freeze-dried single strain of lactobacilli or bifidobacteria, which viability was assessed probing membrane integrity. Present approach and results cannot be generalized to multi-strain products (since FFC would need the development of specific markers, and IFC is label free) nor correlated to plate count methodologies since the measurand is different and based on cellular replication.

## Data availability statement

The raw data supporting the conclusions of this article will be made available by the authors, without undue reservation.

## Author contributions

PJ: Conceptualization, Data curation, Formal analysis, Investigation, Methodology, Validation, Visualization, Writing – original draft, Writing – review & editing. MD: Data curation, Software, Visualization, Writing – review & editing. CM: Formal analysis, Writing – review & editing. SA: Conceptualization, Data curation, Validation, Writing – review & editing. DZ: Investigation, Writing – review & editing, Methodology. DC: Methodology, Writing – review & editing. SG: Methodology, Writing – review & editing. EG: Methodology, Writing – review & editing. J-PW: Supervision, Writing – review & editing. MP: Conceptualization, Methodology, Writing – original draft, Writing – review & editing.
